# Linear Spatial Misregistration Detection and Correction Based on Spectral Unmixing for FAHI Hyperspectral Imagery

**DOI:** 10.3390/s22249932

**Published:** 2022-12-16

**Authors:** Xiangyue Zhang, Xiaoyu Cheng, Tianru Xue, Yueming Wang

**Affiliations:** 1 Key Laboratory of Space Active Opto-Electronics Technology, Shanghai Institute of Technical Physics, Chinese Academy of Sciences, Shanghai 200083, China; 2 University of Chinese Academy of Sciences, Beijing 100049, China; 3 Hangzhou Institute for Advanced Study, University of Chinese Academy of Sciences, Hangzhou 310024, China; 4 Research Center for Intelligent Sensing Systems, Zhejiang Laboratory, Hangzhou 311100, China

**Keywords:** sensor rotation, linear spatial misregistration, hyperspectral unmixing

## Abstract

In push-broom hyperspectral imaging systems, the sensor rotation to the optical plane leads to linear spatial misregistration (LSM) in hyperspectral images (HSIs). To compensate for hardware defects through software, this paper develops four methods to detect LSM in HSIs. Different from traditional methods for grayscale images, the method of fitting the sum of abundance (FSAM) and the method of searching for equal abundance (SEAM) are achieved by hyperspectral unmixing for a selected rectangular transition areas containing an edge, which makes good use of spatial and spectral information. The method based on line detection for band-interleaved-by-line (BIL) images (LDBM) and the method based on the Fourier transform of BIL images (FTBM) aim to characterize the slope of line structure in BIL images and get rid of the dependence on scene and wavelength. A full strategy is detailed from aspects of data selection, LSM detection, and image correction. The full spectrum airborne hyperspectral imager (FAHI) is China’s new generation push-broom scanner. The HSIs obtained by FAHI are tested and analyzed. Experiments on simulation data compare the four proposed methods with traditional methods and prove that FSAM outperforms other methods in terms of accuracy and stability. In experiments on real data, the application of the full strategy on FAHI verifies its effectiveness. This work not only provides reference for other push-broom imagers with similar problems, but also helps to reduce the requirement for hardware calibration.

## 1. Introduction

Hyperspectral imaging combines imaging technology and spectroscopy technology to obtain sets of data cubes composed of cross-track, along-track, and spectral dimensions. The hyperspectral images (HSIs) contain spatial, spectral, and radiation information, which greatly improves the ability to monitor the Earth system and human activities [1]. In particular, the high spectral resolution allows for hundreds of bands to identify ground objects with spectral similarity [2,3,4]. It is important for hyperspectral sensors to capture spectral features accurately, because it will affect hyperspectral image applications [5,6].

**Figure 1:**
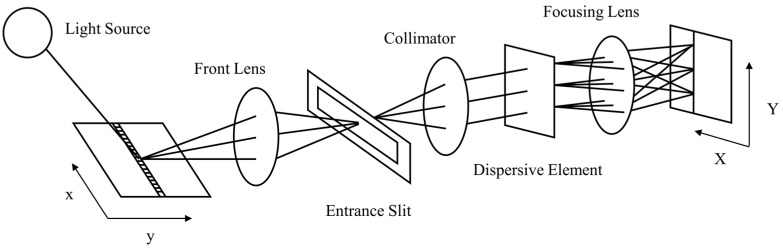
The workflow of push-broom hyperspectral imagers. (x refers to the cross-track direction, and y refers to the along-track direction; X is the spatial dimension in detector, and Y is the spectral dimension).

The hyperspectral imaging system mainly includes a line scanning optical imaging system and a push-broom imaging system. Among them, push-broom imaging is widely used in practice because of its simple structure, high signal-to-noise ratio, and high spectral and spatial resolution [7]. The full spectrum airborne hyperspectral imager (FAHI), China’s new generation push-broom hyperspectral imager, has been applied to civil and environmental monitoring [8,9]. The workflow of a push-broom instrument is shown in Figure 1. Briefly, as the scanner pushes forward along the track, the radiation from substances in a linear field of view is received by the detector array after collimation, dispersion, and convergence of the optical system. Two common optical artifacts occur in this process, which are keystone and smile [10,11]. Keystone is caused by the lateral chromatic aberration of the off-axis dispersive beam exiting from the slit edge. It acts in such a way that a straight line is imaged as a curve for a given pixel in spatial dimensions, known as spatial misregistration. Smile results from the dispersion difference of the dispersive element for the beam emitted from different positions of slit. It leads to the curved line on the image sensor for a given wavelength, known as spectral misregistration. Actually, laboratory measurements are available for keystone and smile optimization, and their values are usually specified in the camera specifications as primary optical indicators [12]. To obtain spectral signatures useful for scientific research, some studies made requirements for keystone and smile. A spectral uncertainty of less than 0.01 of the full width at half maximum throughput of the spectral response function is considered to be necessary, and a maximum level of allowed keystone to be 5% of a pixel is set [13,14].

**Figure 2:**
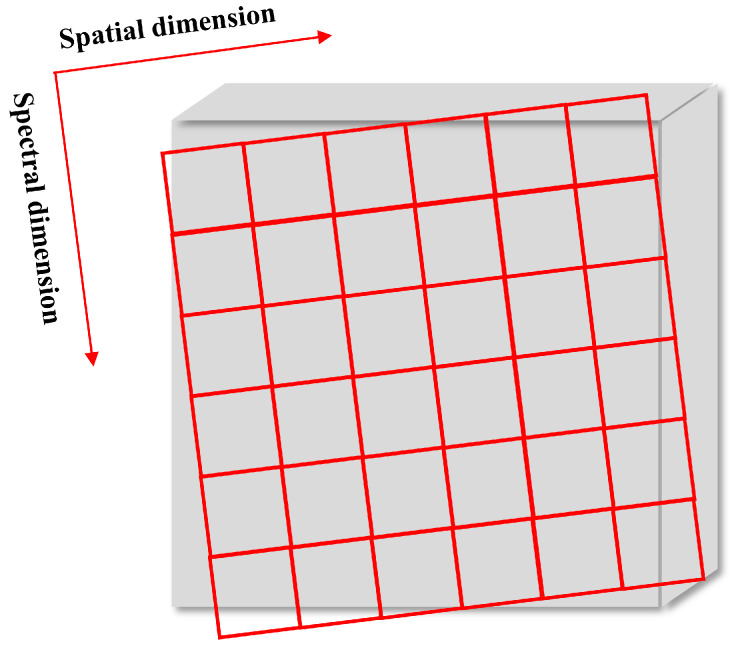
Diagram of detector rotational misalignment.

However, this paper focuses on another image artifact faced by push-broom hyperspectral imagers—linear spatial misregistration (LSM) caused by sensor rotation, which has been neglected in the past. It has been mentioned in previous articles, but not in detail [15]. In addition, LSM was detected in hyperspectral data obtained by Hyperion and SWIR (short wave Infrared) full spectrum imager (SFSI) instruments [6,16]. Figure 2 explains the generation of this artifact, where the gray cube denotes the optical plane, and the red grid denotes the rotational detector array. It refers to the rotational misalignment of the sensor to the optical plane and has nothing to do with the whole optical system. The approximation of a linear function of bands provides a reasonable model for LSM detection and correction in HSIs [6]. There is a problem that keystone also produces spatial misregistration and theoretically affects the detection performance. Considering the following aspects: (1) keystone drives nonlinear spatial misregistration and has a maximum level below 5% of a pixel, as mentioned above; (2) hyperspectral data contain hundreds of bands; and (3) the setting of threshold in our method hinders keystone to some extent, hence, the impact of keystone for LSM detection is considered negligible in our research.

LSM prevents accurate spectrum acquisition. It weakly distorts the spectrum of pixels in the uniform regions and severely distorts the spectrum of pixels in transition areas, which will be verified in our simulation experiments. The distorted spectral curves bring errors to hyperspectral applications, especially to sub-pixel level applications [17,18,19]. In this case, it is necessary to develop LSM detection and correction preprocessing tools to eliminate LSM and recover spectrum, which compensates for the hardware effects though software. In another way, it reduces the requirement for hardware calibration and provides reference for hardware calibration accuracy. In the absence of prior information, such as the spectral signature of a certain substance, the spatial location of an edge is an available feature for subpixel analysis. In past studies, scene-based methods have been proposed for spatial misregistration detection, which are commonly achieved by locating edges at the subpixel level. The correlation-based method is widely used, including the cross-correlation method (CC) and the phase correlation method (PC) [20,21,22,23]. Robert calculated the Pearson correlation coefficient over a user defined sliding correlation window at the most prominent edge and determined the inter-band subpixel shift by interpolating the correlation values [16]. Naoto used the normalized cross-correlation method and the phase correlation method to detect spatial misregistration, and the latter provided better detection performance [6]. Specifically, the phase correlation method determined the peak location of the phase correlation function as misregistration between two bands. Gradient maximization (GM) is also one of the effective ways for subpixel edge detection [24,25], which generally determines the subpixel location by fitting pixels of each row and calculating the maximum gradient. Endice improved it by carrying out a weighted sum to refine each location for spatial misregistration detection [15]. These methods were initially used in grayscale images, so they only exploit the spatial information and ignore spectral information in HSIs. Sometimes, they suffer from instability.

To improve the LSM detection performance, spectral unmixing is adopted for spectral analysis, which has been widely used in subpixel analysis. [26,27,28]. The method of fitting the sum of abundance (FSAM) and the method of searching for equal abundance (SEAM) are developed to detect LSM, which rely on the linear mixture model (LMM) in spectral unmixing and make good use of the spatial and spectral information in HSIs. FSAM is achieved by fitting the sum of abundance of each row in the selected data, and SEAM aims to search for bands with equal abundance for two adjacent pixels. FSAM and SEAM need rectangular transition areas containing clear edges as test subjects. Each side of the edge is composed of pure pixels (endmembers), and the transition area consists of mixed pixels. To get rid of the dependence on scene, the method based on line detection for band-interleaved-by-line (BIL) images (LDBM) and the method based on the Fourier transform of BIL images (FTBM) are produced. They take images in the form of BIL as test data, considering BIL images intuitively reflect the linear change of radiation with wavelength. LDBM directly performs line detection on BIL images, and FTBM characterizes the lines in the frequency domain of BIL images. According to the LSM detection result, cubic spline interpolation is adopted to correct the HSIs. To make it clear, a full three-step strategy is detailed from aspects of data selection, LSM detection, and image correction.

As one of the airborne push-broom imagers, FAHI suffers the problem of sensor rotation due to mechanical instability and hardware adjustment, which leads to varying degrees of LSM in HSIs obtained from each flight mission. In this paper, the hyperspectral data of FAHI is used in our experiments. On the one hand, the performance of the proposed detection methods is tested and compared through simulation experiments. On the other hand, the artifact existing in FAHI is detected, analyzed, and corrected.

The major contributions of this paper are as follows: This paper concentrates on the LSM phenomenon caused by sensor rotation in push-broom hyperspectral imaging systems, proposes novel methods for HSIs to quantify LSM, and details a full strategy to eliminate LSM. The application of the strategy on FAHI verifies its effectiveness. Moreover, the work provides solutions for other push-broom scanners with similar problems and helps to simplify hardware requirements by compensating for hardware effects in software.

## 2. Materials and Methods

**Figure 3:**
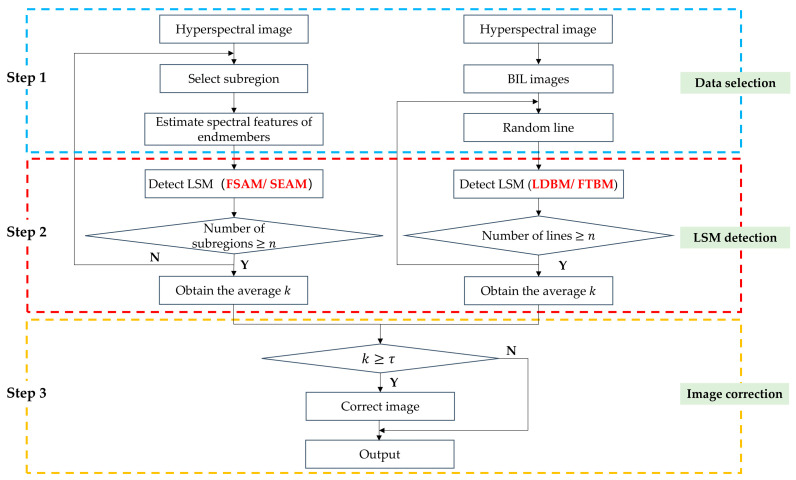
Flowchart of the proposed three-step strategy.

The proposed three-step strategy is used to deal with the spatial misregistration caused by rotational misalignment of the detector, which contains three parts corresponding to data selection, LSM detection, and image correction, as shown in Figure 3.

### 2.1. Data Selection

The removal of LSM should be performed on HSIs without radiometric and geometric corrections since the operation of resampling degrades spectral signatures, which is a common premise. The four proposed detection methods have different requirements for experiment data. FSAM and SEAM depend on the availability of edges in the HSIs, while LDBM and FTBM need to convert HSIs into BIL format. These are described separately in the following paragraphs.

For FSAM and SEAM, subregions containing clear edges crossing the along-track direction are first cropped from HSIs as experiment data. To ensure the accuracy of the subsequent endmember’s extraction, it is necessary to check that each side of the edge is a homogenous area covered by one kind of substance. It is better to choose continuous bands with high signal-to-noise ratio and similar scene contrast, which reduce the computational cost on the one hand and improve the detection accuracy on the other hand. Generally, at least three different subregions are selected in each of the left, center, and right field of view (FOV) to enhance the reliability.

For each subregion, endmembers are then extracted on both sides of the edge to prepare for FSAM and SEAM detection. Considering the complexity of surface distribution and the existence of spectral variability [29,30], an endmember extraction algorithm is not suitable for this work. For one thing, it is not sure whether there are endmembers in the cropped subregions, especially when the spatial resolution is low. For another, the mixed pixels in the transition area may be the result of the complex action of various ground objects, and using endmember extraction methods based on LMM will bring large errors. In this work, regions of interest (ROIs) are selected on both sides of the edge and take the average of the pixel’s spectrum within ROIs as representative endmembers.

For LDBM and FTBM, the hyperspectral image in BIL format is required, where the horizontal direction represents spatial dimension, and the vertical direction represents spectral dimension. LDBM and FTBM utilize the feature that BIL images can reflect the linear change of radiation with wavelength for LSM detection. There are three ways of storing hyperspectral data, including BIL, band sequential format (BSQ), and band-interleaved-by-pixel format (BIP), which can be converted to each other.

### 2.2. LSM Detection

Four methods are developed here for LSM detection. Among them, FSAM and SEAM use vectorized LMM in hyperspectral unmixing and perform abundance analysis to obtain the detected LSM. LDBM and FTBM aim to characterize the slope of the structure in BIL images and convert it to LSM.

#### 2.2.1. Methods Based on Hyperspectral Unmixing

To make good use of spatial and spectral information in HSIs, LMM is improved to vectorized LMM. Based on that, two methods are presented. In this part, vectorized LMM, FSAM, and SEAM are introduced successively.

##### Vectorized LMM

Hyperspectral unmixing aims to decompose mixed pixels into a combination of endmembers, including the process of endmember extraction and abundance estimation [31]. The former extracts the spectra of endmembers from the hyperspectral image, and the latter determines the abundance of each endmember in mixed pixels. The mixing model provides the basis for hyperspectral unmixing, which contains LMM and the nonlinear mixing model (NLMM) [32,33]. In practice, LMM is most broadly used in terms of its simple structure and good performance. LMM uses a linear combination of endmembers weighted by the corresponding abundance to describe pixels.

For a hyperspectral image $Y=\left[y_{1},y_{2},\ldots ,y_{N}\right]\in {\mathbb{R}}^{L\times N}$ with p endmembers, LMM can be expressed as:

(1) Y=EA+n,

where $E=\left[e_{1},e_{2},\ldots ,e_{p}\right]\in {\mathbb{R}}^{L\times p}$ is the endmember matrix, $A=\left[a_{1},a_{2},\ldots ,a_{N}\right]\in {\mathbb{R}}^{p\times N}$ is the abundance matrix, and $n\in {\mathbb{R}}^{L\times N}$ represents the additive noise matrix. The abundance matrix is required to satisfy two constraints, the abundance non-negativity constraint (ANC):

(2) $$a_{i}\geq 0,$$

and the abundance sum-to-one constraint (ASC):

(3) $$1^{T}a_{i}=1,$$

where 1 is a vector of ones, and i=1,…,N.

**Figure 4:**
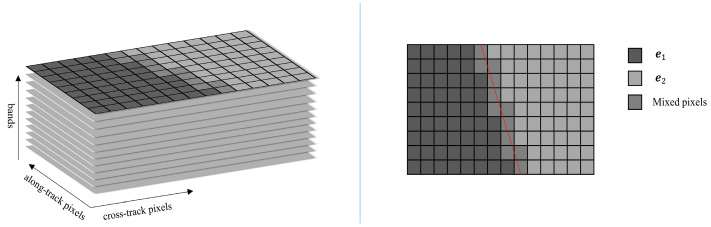
The selected subregion with a crossed edge represented by the red line.

Figure 4 shows a selected subregion and the image of one band, where the red line represents the subpixel edge location. Both sides of the edge consist of pure pixels corresponding to two types of spectral signatures, which are denoted by $e_{1}$ and $e_{2}$. According to LMM, pixels crossed by the edge are mixed pixels formed by linear combination of the two endmembers. If there is no LSM between bands, a given pixel y can be described as:

(4) $$y=\alpha e_{1}+\beta e_{2},$$

where α and β are abundances corresponding to $e_{1}$ and $e_{2}$. However, if there is LSM in the hyperspectral image, the abundances are no longer constant but vary with bands. The given pixel y is formulated as:

(5) $$y=\alpha e_{1}+\beta e_{2},$$

where $\alpha ={\left[\alpha_{1},\alpha_{2},\ldots ,\alpha_{L}\right]}^{T}$ and $\beta ={\left[\beta_{1},\beta_{2},\ldots ,\beta_{L}\right]}^{T}$ are abundance vectors recording the abundance for each band to $e_{1}$ and $e_{2}$, respectively. If $e_{1}$ and $e_{2}$ are known, the abundance vectors can be calculated as:

(6) $$\alpha =\frac{y-e_{2}}{e_{1}-e_{2}},$$

(7) $$\beta =\frac{y-e_{1}}{e_{2}-e_{1}}.$$

According to ASC and ANC in LMM,

(8) $$\alpha_{i}+\beta_{i}=1,\;\;\;\alpha_{i}\geq 0,\;\;\beta_{i}\geq 0,$$

where i=1,…,L. Perform hyperspectral unmixing on all pixels, and a three-dimensional abundance map is obtained for $e_{1}$ or $e_{2}$.

##### FSAM Detection

**Figure 5:**
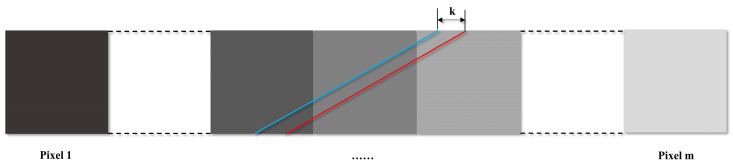
The subpixel edges (represented by red and blue) under two adjacent bands in a row of the abundance map.

FSAM is achieved by fitting the sum of abundance of each row in the selected data. The edge splits each pixel into two parts, where one part is covered by $e_{1}$, and the other part is covered by $e_{2}$. In the abundance map, each pixel can be considered as a square of 1×1, as the pixel value records the area covered by $e_{1}$ or $e_{2}$ ranging from 0 to 1. Figure 5 shows a row of the jth band in the abundance map, containing m pixels. The blue line represents the edge in the jth band, and the red line represents the edge in the (j+1)th band with offset of k pixel from the blue line. Then, the difference ΔS in the area covered by $e_{1}$ or $e_{2}$ in these two bands can be represented by k:

(9) $$\Delta S=k=S_{j+1\_left}-S_{j\_left}=S_{j\_right}-S_{j+1\_right},$$

where $S_{j+1\_left}$ refers to the left area of the red line, $S_{j\_left}$ is the left area of the blue line, is the right area of the blue line, and $S_{j+1\_right}$ refers to the right area of the red line. Use abundance to replace:

(10) $$S_{j+1\_left}=\overset{n}{\underset{i=1}{\sum }}\alpha_{j+1}^{i},$$

(11) $$S_{j\_left}=\overset{n}{\underset{i=1}{\sum }}\alpha_{j}^{i}.$$

where $\alpha_{j}^{i}$ is the abundance of the ith pixel in the jth band for $e_{1}$. Thus, k is given as:

(12) $$k=\overset{n}{\underset{i=1}{\sum }}\alpha_{j+1}^{i}-\overset{n}{\underset{i=1}{\sum }}\alpha_{j}^{i}$$

To reduce the error caused by endmember estimation and noise, calculate S for all bands and obtain $S={\left[S_{1\_left},S_{2\_left},\ldots ,S_{L\_left}\right]}^{T}$. Then, a linear fit to S according to $Z=\hat{k}S+b$ is performed, where $\hat{k}$ is used to denote the estimated inter-band misregistration for this row. The $\hat{k}$ of all rows in the subregion is averaged as the final LSM caused by rotational misalignment.

##### SEAM Detection

For two adjacent pixels in a row, SEAM aims to search for two bands with the same abundance. Specifically, for each row in the selected subregion, a middle band with high signal-to-noise ratio is taken to perform the Sobel operator to enhance the edge. Supposing that the ith pixel obtains the maximum value, the abundance vectors to $e_{1}$ or $e_{2}$ for the *i*th pixel and the (i+1)th pixel are calculated, which correspond to $\alpha^{i}={\left[\alpha_{1}^{i},\alpha_{2}^{i},\ldots ,\alpha_{L}^{i}\right]}^{T}$ and $\alpha^{i+1}={\left[\alpha_{1}^{i+1},\alpha_{2}^{i+1},\ldots ,\alpha_{L}^{i+1}\right]}^{T}$.

For $\alpha_{j}^{i}$ in $\alpha^{i}$, $\alpha_{l}^{i+1}$ could be found in $\alpha^{i+1}$ and satisfies:

(13) $$\left|\alpha_{j}^{i}-\alpha_{l}^{i+1}\right|<\varepsilon$$

where *ε* is a small constant. It is set as 0.005 in our experiments. To improve the robustness, it is necessary to limit the band range:

(14) |j−l|>τ

where τ is an integer. For different band offset, the optimal value of τ is different.

Then, k is given by:

(15) $$k=\frac{1}{\left|j-l\right|}$$

**Figure 6:**
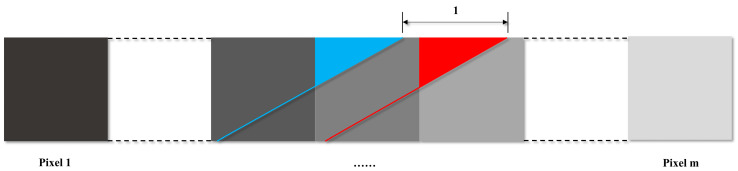
The subpixel edges (represented by red and blue) under two bands with offset of 1 in a row of the abundance map.

Figure 6 depicts this case. The blue line represents the subpixel edge in the jth band of the row, and the red line represents the subpixel edge in the lth band with an offset of 1 to the blue line. The blue area and the red area denote $\alpha_{j}^{i}$ and $\alpha_{l}^{i+1}$—the abundance of the *i*th pixel and the (i+1)th pixel to endmember $e_{1}$ or $e_{2}$.

For the ith pixel, multiple k can be obtained. The average of the multiple k as an offset for this row and the average of offsets for all rows are taken as the final LSM for the subregion.

#### 2.2.2. Methods Based on BIL Images

Though FSAM and SEAM take advantage of spatial and spectral information in HSIs, they rely on the availability of edges in the image between homogeneous areas. To get rid of the dependence on scene, two methods based on slope detection are proposed, which are simple to implement on BIL images.

##### LDBM Detection

**Figure 7:**
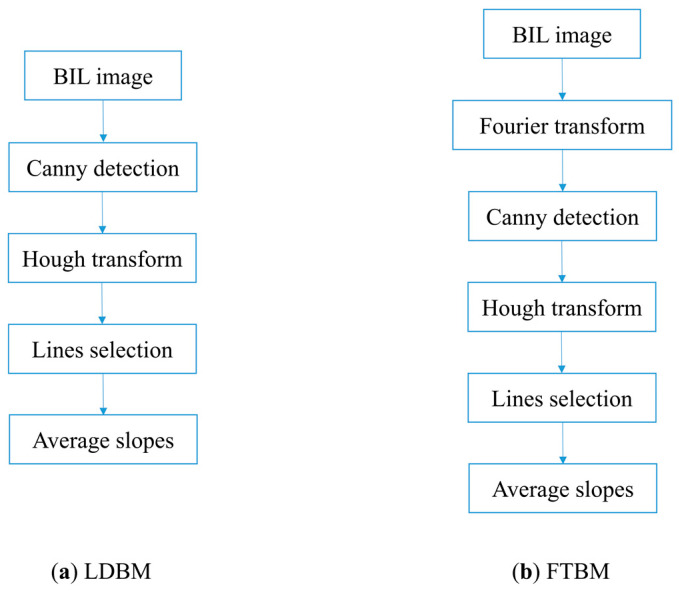
Flowcharts of (**a**) LDBM method and (**b**) FTBM method.

The BIL image contains both spatial and spectral information in a linear field of view. The LSM phenomenon is intuitively reflected in BIL images, leading the texture to skew to a certain angle. Thus, an idea for LSM detection is to perform line detection on the BIL image, and the slope of the detected line is taken as the LSM result. The flowchart of LDBM is shown in Figure 7a.

Canny operator is widely used for edge detection because of its high precision. Due to the influence of spatial resolution and the existence of mixed pixels, the edges in BIL image are blurred. In LDBM, for a BIL image, the canny operator is first adopted to enhance the edges. Then, the Hough transform is used to recognize the line features. Based on the voting mechanism, the lines corresponding to the top n voting peaks are selected as effective detection results. Parameter n is set as 3, 5, or 7 in our experiments. The coordinates of the head and end points of each line are given by the Hough transform, through which the slope of the line can be calculated. Finally, take average of the slopes of the effective lines as LSM.

##### FTBM Detection

The rotation component of an image can be clearly displayed in the frequency domain, specifically, presenting strips inclined at a certain angle. So FTBM is determined, which tries to recognize the slope in the Fourier transform of BIL images. The flowchart of FTBM is shown in Figure 7b.

For a BIL image, fast Fourier transform is first needed to convert it to the frequency domain. Then, the canny operator is applied to extract the edge information. The continuous line structure is characterized by the Hough transform. The lines with the first n (3, 5, or 7) voting peaks are selected. Finally, average the slopes of the selected lines as the final LSM.

In LDBM and FTBM, the canny operator, Hough transform, and Fourier transform are realized through functions in MATLAB 2019a, which make the methods simple and fast to achieve. FSAM and SEAM are more complicated to conduct than LDBM and FTBM. But FSAM has the potential to be applied to nonlinear spatial misregistration detection; the other three are mainly used for linear detection.

### 2.3. Image Correction

After detecting LSM, the image should be corrected according to detected result. As the smaller the k and the larger the detection error, a threshold is set to compare with k. If k is less than the threshold, the effect of LSM is ignored. Contrarily, the hyperspectral image needs to be corrected. Based on the detection results in the simulation experiments, the threshold is set to be 0.005.

The correction method is based on cubic interpolation because of its good trade-off between smoothness and shape preservation [34]. Specifically, a reference band is first selected, generally in the middle band with high signal-to-noise ratio. Then, the offset of other bands to the reference band is calculated:

(16) $$offset_{j}=k\left(j-i\right),$$

where the reference band is the ith band, and $offset_{j}$ is the offset of the jth band to the ith band. Finally, perform interpolation on each band to correct the image.

## 3. Results and Discussion

In this Section, experiments on simulation data and real data are conducted to assess the performance of the proposed methods and the three-step strategy. In experiments on simulation data, the accuracy and the adaptability to different spatial and spectral resolutions of FSAM, SEAM, LDBM, and FTBM are mainly tested with PC, CC, and GM as comparison. In experiments on real data, the full strategy is applied to real data of FAHI, aiming to analyze the artifact in FAHI and prove its feasibility and effectiveness.

### 3.1. Experimental Data

**Figure 8:**
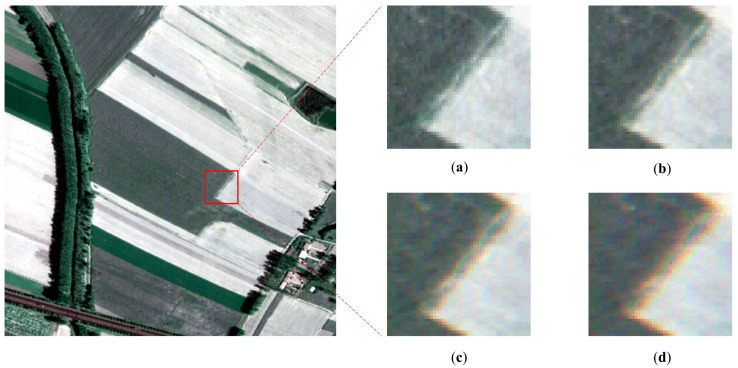
Pseudo-color sub-images with k of (**a**) 0; (**b**) 0.01; (**c**) 0.03; (**d**) 0.05.

**Figure 9:**
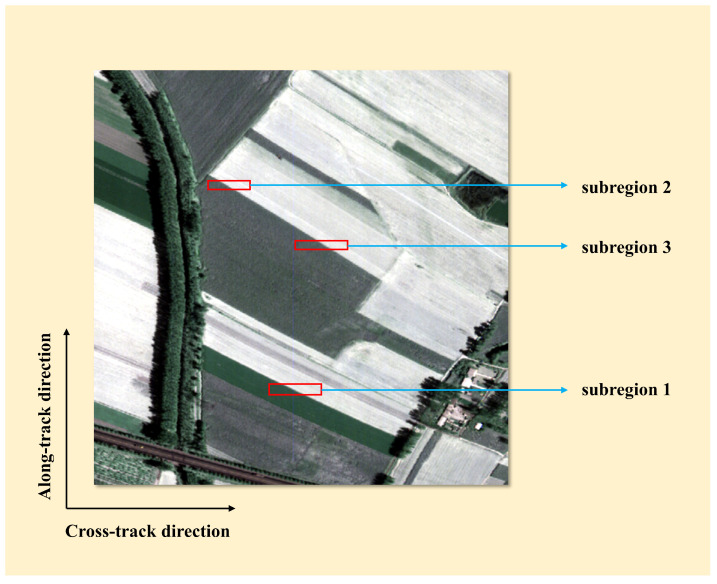
Pseudo-color image of the selected subregions.

The hyperspectral data obtained by FAHI is used in our experiments [35,36]. To evaluate the detection ability for LSM of the proposed methods, we conducted experiments on simulation data. Since whether LSM exists or its value cannot be determined in advance, a sub-image with a detected result less than the threshold was selected as original data. Its size is 500 × 500, and it contains 90 bands covering a spectral range from 1500 to 1800 nm. The spatial resolution is 0.5 m, and the spectral resolution is 3.2 nm. To induce LSM, the band offsets of 0.01, 0.03, and 0.05 of a pixel were set on it using bilinear interpolation functions, respectively, with the first band as the reference band. Then, three simulated datasets were obtained. Figure 8 shows the selected sub-image and the local comparison with different offsets. The larger the offset, the more obvious the artifact. Finally, three subregions containing clear edges were cropped from simulated datasets for LSM detection, as shown in Figure 9.

To verify the effectiveness of the full strategy, experiments were carried out on HSIs of FAHI. For each hyperspectral image, three subregions in each of the left, middle, and right FOV were selected to enhance the reliability of the detection results. The results of an image were presented.

### 3.2. Evaluation Metrics

To evaluate the LSM detection performance of the proposed algorithms, the following two metrics are used:

1. Absolute error (AE):

(17) $$\mathrm{AE}=\left|k-\bar{k}\right|,$$

where $\bar{k}$ is the real offset, and k is the detected offset. AE directly reflects the detection error.

2. Mean root mean square error (MRMSE):

(18) $$\mathrm{MRMSE}=\frac{1}{r}\overset{r}{\underset{i=1}{\sum }}\sqrt{\frac{norm{\left(y_{i}-{\bar{y}}_{i}\right)}^{2}}{L}},$$

where ${\bar{y}}_{i}$ refers to the fitting vector of all bands in the ith row, $y_{i}$ refers to the detected vector of all bands, L is the number of bands, and r is the number of rows. MRMSE indicates the stability of the test algorithm. The smaller the values are of AE and MRMSE, the better the detection results are.

To measure the improvement of the image quality after LSM correction, the following three metrics are calculated [37,38]:

1. Peak signal to noise ratio (PSNR):

(19) $$\mathrm{PSNR}=20\times log_{10}\left(\frac{MAX}{MSE}\right),$$

where MAX is the maximum value of the image, and MSE is the mean square error of two images. PSNR is the most widely used image quality evaluation indicator based on the error between corresponding pixels.

2. Structural similarity (SSIM):

(20) $$\mathrm{SSIM}=\frac{2\mu_{x}\mu_{y}+C_{1}}{\mu_{x}^{2}+\mu_{y}^{2}+C_{1}}\cdot \frac{2\delta_{xy}+C_{2}}{\delta_{x}^{2}+\delta_{y}^{2}+C_{2}},$$

where $C_{1}$ and $C_{2}$ are constants, $\mu_{x}$ and $\mu_{y}$ are the mean gray values of two images, $\delta_{x}$ and $\delta_{y}\;$are standard deviations of two images, and $\delta_{xy}$ is the covariance. SSIM measures the image similarity from brightness, contrast, and structure.

3. Spectral angle mapper (SAM):

(21) $$\mathrm{SAM}=cos^{-1}\frac{x^{T}y}{{\left(x^{T}x\right)}^{1/2}{\left(y^{T}y\right)}^{1/2}},$$

where x and y correspond to the spectral vectors of pixels at the same location in two hyperspectral images. SAM is an important indicator for measuring changes in spectral features, since the spectra determine the identification of substances. The larger the values are of PSNR and SSIM and the smaller the value of SAM, the better the image quality is.

### 3.3. Experiments on Simulation Data

In this section, four sets of experiments are carried out, aiming at comparing the LSM detection performance of all test algorithms, measuring the change of image quality after LSM correction, testing the adaptability to HSIs with different spatial resolution of the proposed methods, and testing the adaptability to HSIs with different spectral resolution of the proposed methods, respectively.

#### 3.3.1. Performance Comparison of Detection Methods

Experiments on simulation data are conducted to assess the performance of the proposed FSAM, SEAM, LDBM, and FTBM with PC, CC, and GM as comparisons. All test methods are performed on the three subregions, as shown in Figure 9, to detect the LSM. The results of detected k, AE, and MRMSE are shown in Table 1. Since SEAM, LDBM, and FTBM do not fit all bands, their MRMSE cannot be calculated; only k and AE are available.

From the experimental results: (1) for each subregion, the values of AE obtained by all test algorithms are all within 0.01; (2) for each subregion, compared with SEAM and FTBM, the proposed FSAM and LDBM obtain lower values of AE, with the lowest for FSAM, which means they have higher detection accuracy; (3) during the experiments, it is found that SEAM and FTBM are less stable as their detection performance overly depends on parameter adjustment, such as an angle range, that is very close to the real situation and needs to be set in the Hough transform function in LDBM and FTBM to maintain a good detection performance; (4) for each subregion, in comparison methods, GM provides relatively lower values of AE, indicating it brings smaller detection errors compared to CC and PC; (5) the MRMSE results exhibit a significant difference for each subregion, and this demonstrates the varying degrees of stability of the algorithms; (6) though GM obtains lower AE, its MRMSE results are highest for different levels of the band shift, showing its sensitivity to noise; (7) FSAM obtains the lowest MRMSE, so it is more stable compared to CC, PC, and GM; (8) compared with other methods (including CC, PC, GM, SEAM, LDBM, and FTBM), FSAM shows a smaller detection error and better stability and is more suitable for LSM detection; (9) the consistency of AE and MRMSE for the three subregions also verifies the capability of FSAM to detect LSM; (10) in addition, it seems that subregion1 obtains larger MRMSE, and subregion3 obtains smaller MRMSE. It is related to the complex distribution of vegetation areas such as different vegetation densities.

In conclusion, the proposed FSAM outperforms other methods (including CC, PC, GM, SEAM, LDBM, and FTBM) in terms of detection error and stability, and the detection error of FSAM is within 0.005.

#### 3.3.2. Improvement of Image Quality

**Figure 10:**
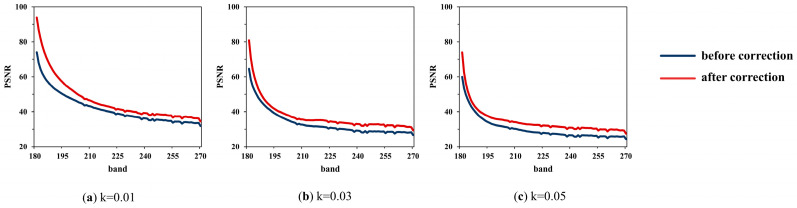
PSNR of each band between original image and images with k of (**a**) 0.01; (**b**) 0.03; and (**c**) 0.05.

**Figure 11:**
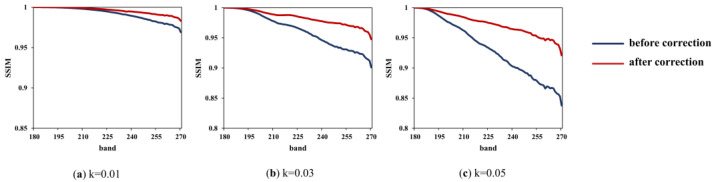
SSIM of each band between original image and images with k of (**a**) 0.01; (**b**) 0.03; and (**c**) 0.05.

**Figure 12:**
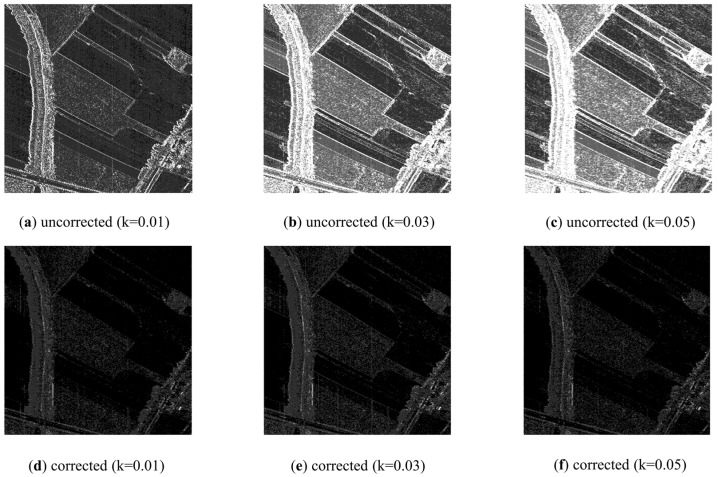
SAM between original image and the (**a**) uncorrected image with k of 0.01; (**b**) uncorrected image with k of 0.03; (**c**) uncorrected image with k of 0.05; (**d**) corrected image with k of 0.01; (**e**) corrected image with *k* of 0.03; (**f**) corrected image with *k* of 0.05.

The average results of the three subregions obtained by FSAM are used to correct the sub-image, which are 0.0098, 0.0302, and 0.0504. To measure the improvement of the image quality after LSM correction, PSNR, SSIM, and SAM are calculated, and the results are shown in Figure 10, Figure 11 and Figure 12.

From the results, the impact of LSM on image quality is quantified: (1) for a certain offset, the farther away it is from the reference band, the worse the band quality is; (2) for a different offset, the larger the offset is, the worse the image quality is; (3) for offsets of 0.01, 0.03, and 0.05, PSNR decreases to 31.8, 26.6, and 24.4, respectively; SSIM decreases to 0.97, 0.90, and 0.84, respectively; (4) SAM increases significantly, which means the spectra deviate from the ground truth; (5) results of SAM also show that the spectral distortion in the edge is larger than that in the homogeneous region.

After LSM correction, the image quality is improved: (1) for a certain offset, PSNR, SSIM, and SAM are all improved compared with those before LSM correction; (2) for offsets of 0.01, 0.03, and 0.05, PSNR of the last band increased to 34.4, 29.4, and 27.5, respectively; SSIM of the last band increases to 0.98, 0.95, and 0.92, respectively; (3) SAM decreases greatly, thus the spectral curves move closer to the real curves; (4) on the whole, the larger the offset, the more the image quality is improved; (5) though the image is recovered, the image with a large offset is still inferior to the image with a small offset.

In conclusion, LSM degrades hyperspectral quality, and the proposed detection and correction is effective for dealing with the problem, as it improves the image quality and brings recovered spectral features.

#### 3.3.3. Adaptability to HSIs with Different Spatial Resolution

To verify the adaptability of the proposed methods to hyperspectral data with different spatial resolution, experimental data with spatial resolution of 1 m and 2 m are obtained by applying pixel merging to the original data [39]. Then, the inter-band offset is set to 0.01, 0.03, and 0.05 of a pixel. The detection results of all test methods on subregion 1, subregion 2, and subregion 3 are shown in Table 2 and Table 3.

From the experimental results: (1) for each subregion, the values of AE obtained by all test algorithms remain below 0.01, among which, FSAM offers lower values of AE; (2) compared to the results in Table 1, with the decrease in spatial resolution, AE of CC, PC, GM, FSAM, and SEAM show weak fluctuation, indicating the methods keep their performance for LSM detection; (3) relatively, AE of LDBM and FTBM have clear fluctuation, indicating they are affected by spatial resolution, as the lower the spatial resolution is, the blurrier the edges in BIL images are; (4) for each subregion, GM obtains the highest values of MRMSE, and FSAM and PC obtain lower values of MRMSE, which shows that FSAM and PC are more stable than GM and CC; (5) compared to the results in Table 1, with the decrease in spatial resolution, MRMSE values of test algorithms decrease significantly, and it is mainly because the images with high spatial resolution can display rich details of ground objects; (6) the MRMSE results also indicate that the algorithms have different stability at different levels of spatial resolution; (7) FSAM keeps lower MRMSE to HSIs at different spatial resolution, which shows the FSAM is more robust to spatial resolution changes compared with CC, PC, and GM.

In conclusion, the spatial resolution mainly affects the detection accuracy of LDBM and FTBM, and the algorithm stability of CC, PC, GM, and FSAM. FSAM shows better adaptability to HSIs with spatial resolution from 0.5 m to 2 m in terms of detection accuracy and stability, and the absolute detection error is kept within 0.005.

#### 3.3.4. Adaptability to HSIs with Different Spectral Resolution

To verify the adaptability of the proposed methods to hyperspectral data with different spectral resolution, experimental data with spectral resolution of 6.4nm and 9.6nm are obtained by applying band merging to the original data [39]. Then, the inter-band offset is set to 0.01, 0.03, and 0.05 of a pixel. The detection results of all test methods on subregion 1, subregion 2, and subregion 3 are shown in Table 4 and Table 5.

From the experimental results: (1) for each subregion, the values of AE obtained by all test algorithms are below 0.01, and FSAM still provides detection error less than 0.005; (2) compared to the results in Table 1, with the decrease in spectral resolution, AE of the test algorithms shows an increasing tendency in fluctuation overall, demonstrating the detection accuracy of most algorithms is reduced; (3) relatively, the AE values obtained by FSAM, PC, and GM show little change, which indicates that FSAM keeps better ability to detect LSM at the spectral resolution of 6.4nm and 9.6nm; (4) AE of LDBM, FTBM, and CC increase significantly, which represents they are easily affected by spectral resolution; (5) for each subregion, GM obtains higher values of MRMSE, and FSAM still keeps lower values of MRMSE, showing the good stability of FSAM; (6) compared to the results in Table 1, with the decrease in spectral resolution, MRMSE shows slight fluctuation, meaning the stability of CC, PC, GM, and FSAM remains steady with the change of spectral resolution.

It is concluded that the spectral resolution affects the detection accuracy of most test methods. The higher the spectral resolution is, the more number of bands there are, and the higher the detection accuracy. Considering detection error and algorithm stability, FSAM provides better adaptability to HSIs with spectral resolution from 3.2 nm and 9.6 nm compared with other methods, and the absolute detection error remains within 0.005.

### 3.4. Experiments on Real Data

In this section, the results of a hyperspectral image obtained by FAHI are present. Three subregions are selected in each of the left, middle, and right FOV of the image, and the proposed FSAM is utilized to detect LSM on the subregions. The results of detected k are shown in Table 6.

**Figure 13:**
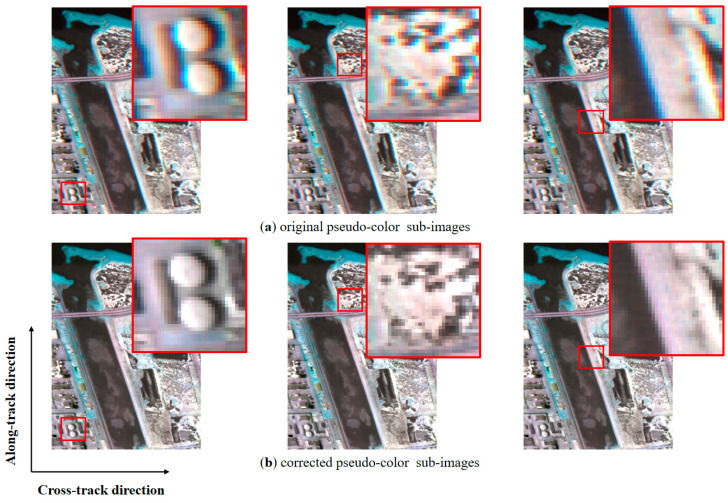
Comparison between (**a**) original sub-images and (**b**) corrected sub-images of buildings, plants, and seaside areas.

**Figure 14:**
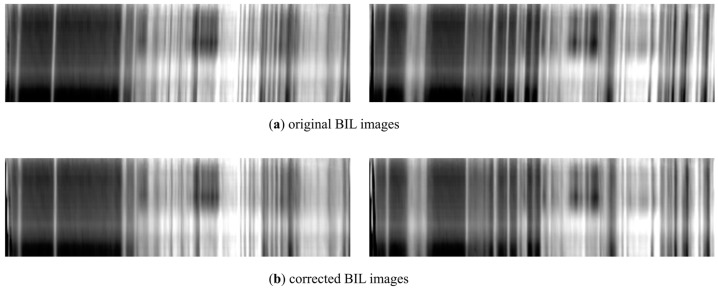
Comparison between (**a**) original BIL images and (**b**) corrected BIL images of two lines with spectral range from 8.3 μm to 10.5 μm.

Results detected for all subregions are all around 0.03 with slight fluctuation (Table 6), which demonstrates the sensor rotation existing in FAHI instrument. The average of the results is used to correct the image, and qualitative analysis is implemented here. The buildings, plants, and seaside areas before and after correction are compared in Figure 13. In the original sub-images, there is noticeable ghosting phenomenon caused by spatial misregistration, especially in the transition areas involving two or more substances. In the corrected sub-images, this phenomenon is greatly reduced, and the transition regions become sharp and clear. The image in BIL format also reflects the change before and after correction, where the horizontal direction represents spatial dimension, and the vertical direction represents spectral dimension. The BIL images of the selected two lines are shown in Figure 14. As can be seen, there are stripes inclined at a certain angle to the spectral dimension in the original images, and this angle is significantly reduced after LSM correction. The application on FAHI proves the effectiveness and the feasibility of the full strategy.

## 4. Discussion

Overall, the results of the selected subregions show consistency, which enhances the reliability of detection. For the proposed methods, FSAM and SEAM are more complex to conduct, since they depend on the availability of edges in the homogenous area. LDBM and FTBM are easier to perform, as they are based on BIL images. From the scope of application, FSAM calculates the offset for each band and can be used for nonlinear detection, while the other three are only used for LSM detection. In terms of detection accuracy, FSAM and LDBM perform better than SEAM and FTBM. SEAM searches for bands with equal abundance, thus it has a high requirement on spectral unmixing. The line structure in the frequency domain after the Fourier transform is nearly covered by other complex frequency components, resulting in the large error in FTBM. In terms of stability, it is found that the performance of SEAM, LDBM, and FTBM is easily affected by parameter setting. In SEAM, the parameter τ is set differently according to the simulated inter-band shift. A rotation angle range that is close to the real value needs to be set in LDBM and FTBM, and a range less than 2° achieves better accuracy. For comparison methods, GM offers small absolute error but poor stability, while CC and PC balance on both. Though CC, PC, and GM obtain the offset for each band, it is inferred that CC and PC can achieve high precision on nonlinear spatial misregistration detection.

In addition, LDBM and FTBM are more sensitive to the decrease in spatial resolution, owing to the blurred edge information in BIL images at low spatial resolution. The other methods keep their accuracy and obtain better fitting stability, which is mainly because the decreased spatial resolution leads to the loss of detailed information. All methods demand high spectral resolution to realize good performance. The higher the spectral resolution, the more number of bands there are, and the higher the detection accuracy is.

Considering accuracy and stability, FSAM performs better in LSM detection compared to other methods. In the field of linear detection, FSAM, LDBM, and GM can be selected for adoption. In nonlinear detection, FSAM, CC, and PC show their advantages. Moreover, for HSIs with spatial resolution and spectral resolution set in our experiments, the detection error of all methods is less than 0.01 of a pixel, especially if that of FSAM is less than 0.005 of a pixel, which simplifies the requirement for lab calibration and provides reference value for the calibration accuracy.

## 5. Conclusions

In push-broom hyperspectral imagers, the sensor rotation to optical axis leads to LSM in HSIs. This paper develops methods (including FSAM, SEAM, LDBM, and FTBM) to detect LSM and details a full strategy to eliminate LSM. It facilitates the analysis of the sensor rotation degree in push-broom scanners and, more importantly, reduces the hardware demand by compensating for hardware effects in software. Specifically, the experimental data is first selected. For FSAM and SEAM, subregions with distinct edges in each of the left, center, and right FOV are selected, and representative spectrum are extracted as endmembers for each subregion. For LDBM and FTBM, the hyperspectral image needs to be converted to a BIL image as input. Then, the detection method is applied to evaluate LSM. FSAM and SEAM conduct abundance analysis for the abundance map after spectral unmixing, while LDBM and FTBM aim to recognize the slope of the line structure. Finally, cubic interpolation is performed on each band to correct the image according to the detected result.

The proposed methods are tested by experiments on simulation data with CC, PC, and GM as comparison. For HSIs with spatial resolution of 0.5 m, 1 m, and 2 m, and spectral resolution of 3.2 nm, 6.4 nm, and 9.6 nm, all methods ensure that the absolute detection error is less than 0.01. Though FSAM is more complicated to implement, it outperforms other methods in terms of accuracy and stability with absolute error less than 0.005 and shows good adaptability to the change of spatial resolution and spectral resolution. The sensor rotation existing in FAHI is detected through the proposed strategy in experiments on real data, and the image is effectively calibrated, which provides reference for other push-broom hyperspectral instruments with similar problems.

Though several methods have been proposed to detect LSM, it still leaves much to be desired. The algorithm accuracy determines the application value. Under the premise of accuracy improvement of FSAM, nonlinear spatial misregistration detection of band-by-band will contribute to the joint analysis of keystone and sensor rotation.

## Figures and Tables

**Table 1:** The results of all test methods on subregion 1, subregion 2, and subregion 3.

**subregion 1**									
	**k**			**AE**			**MRMSE**		
	**0.01**	**0.03**	**0.05**	**0.01**	**0.03**	**0.05**	**0.01**	**0.03**	**0.05**
CC	0.0106	0.0342	0.0501	0.0006	0.0042	0.0001	0.2869	0.2724	0.2571
PC	0.0094	0.0267	0.0447	0.0006	0.0033	0.0053	0.2872	0.3022	0.3129
GM	0.0113	0.0306	0.0507	0.0013	0.0006	0.0007	0.5012	0.4776	0.5105
FSAM	0.0104	0.0303	0.0507	0.0004	0.0003	0.0007	0.1601	0.1494	0.1490
SEAM	0.0148	0.0281	0.0533	0.0048	0.0019	0.0033	—		
LDBM	0.0107	0.0313	0.0527	0.0007	0.0013	0.0027			
FTBM	0.0120	0.0316	0.0562	0.0020	0.0016	0.0062			
**subregion 2**									
	**k**			**AE**			**MRMSE**		
	**0.01**	**0.03**	**0.05**	**0.01**	**0.03**	**0.05**	**0.01**	**0.03**	**0.05**
CC	0.0088	0.0330	0.0497	0.0012	0.0030	0.0003	0.1819	0.2024	0.2075
PC	0.0101	0.0290	0.0483	0.0001	0.0010	0.0017	0.1469	0.1469	0.1482
GM	0.0105	0.0304	0.0502	0.0005	0.0004	0.0002	0.3754	0.3836	0.3876
FSAM	0.0101	0.0309	0.0511	0.0001	0.0009	0.0011	0.1217	0.1189	0.1206
SEAM	0.0143	0.0288	0.0515	0.0043	0.0012	0.0015	—		
LDBM	0.0111	0.0303	0.0513	0.0011	0.0003	0.0013			
FTBM	0.0077	0.0331	0.0536	0.0023	0.0031	0.0036			
**subregion 3**									
	**k**			**AE**			**MRMSE**		
	**0.01**	**0.03**	**0.05**	**0.01**	**0.03**	**0.05**	**0.01**	**0.03**	**0.05**
CC	0.0083	0.0330	0.0501	0.0017	0.0030	0.0001	0.1354	0.1665	0.1815
PC	0.0097	0.0287	0.0479	0.0003	0.0013	0.0021	0.1595	0.1577	0.1586
GM	0.0099	0.0295	0.0496	0.0001	0.0005	0.0004	0.3312	0.3330	0.3270
FSAM	0.0088	0.0295	0.0495	0.0012	0.0005	0.0005	0.1200	0.1397	0.1433
SEAM	0.0146	0.0277	0.0490	0.0046	0.0023	0.0010	—		
LDBM	0.0106	0.0302	0.0522	0.0006	0.0002	0.0022			
FTBM	0.0065	0.0320	0.0526	0.0035	0.0020	0.0026			

CC, cross-correlation; PC, phase correlation; GM, gradient maximization; FSAM, the proposed method.

**Table 2:** The results of all test methods on subregion 1, subregion 2, and subregion 3 with spatial resolution of 1 m.

**subregion 1**									
	**k**			**AE**			**MRMSE**		
	**0.01**	**0.03**	**0.05**	**0.01**	**0.03**	**0.05**	**0.01**	**0.03**	**0.05**
CC	0.0088	0.0335	0.0501	0.0012	0.0035	0.0001	0.1706	0.1891	0.1923
PC	0.0105	0.0285	0.0466	0.0005	0.0015	0.0034	0.0893	0.1051	0.0943
GM	0.0128	0.0314	0.0510	0.0028	0.0014	0.0010	0.1824	0.2030	0.1835
FSAM	0.0106	0.0302	0.0497	0.0006	0.0002	0.0003	0.1089	0.0916	0.0856
SEAM	0.0130	0.0309	0.0528	0.0030	0.0009	0.0028	—		
LDBM	0.0123	0.0312	0.0518	0.0023	0.0012	0.0018			
FTBM	0.0062	0.0265	0.0491	0.0038	0.0035	0.0009			
**subregion 2**									
	**k**			**AE**			**MRMSE**		
	**0.01**	**0.03**	**0.05**	**0.01**	**0.03**	**0.05**	**0.01**	**0.03**	**0.05**
CC	0.0079	0.0328	0.0497	0.0021	0.0028	0.0003	0.0918	0.1164	0.1546
PC	0.0097	0.0275	0.0456	0.0003	0.0025	0.0044	0.0823	0.0830	0.0876
GM	0.0125	0.0309	0.0499	0.0025	0.0009	0.0001	0.1341	0.1643	0.1667
FSAM	0.0102	0.0293	0.0477	0.0002	0.0007	0.0023	0.0998	0.0809	0.0925
SEAM	0.0133	0.0294	0.0520	0.0033	0.0006	0.0020	—		
LDBM	0.0144	0.0297	0.0520	0.0044	0.0003	0.0020			
FTBM	0.0051	0.0239	0.0476	0.0049	0.0061	0.0024			
**subregion 3**									
	**k**			**AE**			**MRMSE**		
	**0.01**	**0.03**	**0.05**	**0.01**	**0.03**	**0.05**	**0.01**	**0.03**	**0.05**
CC	0.0083	0.0330	0.0501	0.0017	0.0030	0.0001	0.0809	0.1093	0.1538
PC	0.0103	0.0287	0.0472	0.0003	0.0013	0.0028	0.0790	0.0800	0.0915
GM	0.0127	0.0308	0.0502	0.0027	0.0008	0.0002	0.1565	0.1791	0.1855
FSAM	0.0099	0.0291	0.0497	0.0001	0.0009	0.0003	0.0867	0.0811	0.1247
SEAM	0.0141	0.0271	0.0458	0.0041	0.0029	0.0042	—		
LDBM	0.0117	0.0315	0.0523	0.0017	0.0015	0.0023			
FTBM	0.0038	0.0262	0.0517	0.0062	0.0038	0.0017			

**Table 3:** The results of all test methods on subregion 1, subregion 2, and subregion 3 with spatial resolution of 2 m.

**subregion 1**									
	**k**			**AE**			**MRMSE**		
	**0.01**	**0.03**	**0.05**	**0.01**	**0.03**	**0.05**	**0.01**	**0.03**	**0.05**
CC	0.0084	0.0330	0.0497	0.0016	0.0030	0.0003	0.1286	0.1454	0.1757
PC	0.0106	0.0276	0.0460	0.0006	0.0024	0.0040	0.0560	0.0695	0.0666
GM	0.0131	0.0313	0.0515	0.0031	0.0013	0.0015	0.1611	0.1631	0.1566
FSAM	0.0101	0.0295	0.0501	0.0001	0.0005	0.0001	0.0787	0.0627	0.0642
SEAM	0.0140	0.0303	0.0505	0.0040	0.0003	0.0005	—		
LDBM	0.0086	0.0326	0.0524	0.0014	0.0026	0.0024			
FTBM	0.0041	0.0233	0.0480	0.0059	0.0067	0.0020			
**subregion 2**									
	**k**			**AE**			**MRMSE**		
	**0.01**	**0.03**	**0.05**	**0.01**	**0.03**	**0.05**	**0.01**	**0.03**	**0.05**
CC	0.0084	0.0331	0.0501	0.0016	0.0031	0.0001	0.0805	0.0963	0.1519
PC	0.0105	0.0270	0.0442	0.0005	0.0030	0.0058	0.0515	0.0547	0.0569
GM	0.0123	0.0306	0.0502	0.0023	0.0006	0.0002	0.1028	0.1307	0.1469
FSAM	0.0101	0.0314	0.0497	0.0001	0.0014	0.0003	0.0951	0.0783	0.0987
SEAM	0.0123	0.0277	0.0539	0.0023	0.0023	0.0039	—		
LDBM	0.0128	0.0323	0.0533	0.0028	0.0023	0.0033			
FTBM	0.0039	0.0266	0.0471	0.0061	0.0034	0.0029			
**subregion 3**									
	**k**			**AE**			**MRMSE**		
	**0.01**	**0.03**	**0.05**	**0.01**	**0.03**	**0.05**	**0.01**	**0.03**	**0.05**
CC	0.0086	0.0329	0.0491	0.0014	0.0029	0.0009	0.0650	0.0845	0.2027
PC	0.0105	0.0289	0.0476	0.0005	0.0011	0.0024	0.0509	0.0570	0.0614
GM	0.0131	0.0309	0.0504	0.0031	0.0009	0.0004	0.1471	0.1734	0.1776
FSAM	0.0098	0.0308	0.0503	0.0002	0.0008	0.0003	0.0528	0.0658	0.0890
SEAM	0.0142	0.0303	0.0465	0.0042	0.0003	0.0035	—		
LDBM	0.0121	0.0313	0.0534	0.0021	0.0013	0.0034			
FTBM	0.0063	0.0271	0.0583	0.0037	0.0029	0.0083			

**Table 4:** The results of all test methods on subregion 1, subregion 2, and subregion 3 with spectral resolution of 6.4 nm.

**subregion 1**									
	**k**			**AE**			**MRMSE**		
	**0.01**	**0.03**	**0.05**	**0.01**	**0.03**	**0.05**	**0.01**	**0.03**	**0.05**
CC	0.0102	0.0350	0.0594	0.0002	0.0050	0.0094	0.1984	0.2627	0.2548
PC	0.0126	0.0313	0.0503	0.0026	0.0013	0.0003	0.1809	0.2267	0.2201
GM	0.0142	0.0339	0.0537	0.0042	0.0039	0.0037	0.4753	0.5095	0.4726
FSAM	0.0095	0.0305	0.0494	0.0005	0.0005	0.0006	0.1163	0.1254	0.1186
SEAM	0.0131	0.0323	0.0528	0.0031	0.0023	0.0028	—		
LDBM	0.0126	0.0247	0.0489	0.0026	0.0053	0.0011			
FTBM	0.0135	0.0244	0.0514	0.0035	0.0056	0.0014			
**subregion 2**									
	**k**			**AE**			**MRMSE**		
	**0.01**	**0.03**	**0.05**	**0.01**	**0.03**	**0.05**	**0.01**	**0.03**	**0.05**
CC	0.0071	0.0318	0.0581	0.0029	0.0018	0.0081	0.1294	0.2217	0.1603
PC	0.0107	0.0288	0.0471	0.0007	0.0012	0.0029	0.1907	0.1877	0.1794
GM	0.0093	0.0286	0.0492	0.0007	0.0014	0.0008	0.3034	0.3235	0.2795
FSAM	0.0094	0.0304	0.0526	0.0006	0.0004	0.0026	0.1370	0.1336	0.1438
SEAM	0.0055	0.0287	0.0505	0.0045	0.0013	0.0005	—		
LDBM	0.0162	0.0245	0.0483	0.0062	0.0055	0.0017			
FTBM	0.0121	0.0244	0.0465	0.0021	0.0056	0.0035			
**subregion 3**									
	**k**			**AE**			**MRMSE**		
	**0.01**	**0.03**	**0.05**	**0.01**	**0.03**	**0.05**	**0.01**	**0.03**	**0.05**
CC	0.0070	0.0323	0.0577	0.0030	0.0023	0.0077	0.1447	0.1992	0.1952
PC	0.0094	0.0282	0.0474	0.0006	0.0018	0.0026	0.2135	0.2204	0.2103
GM	0.0112	0.0312	0.0519	0.0012	0.0012	0.0019	0.3372	0.3713	0.3517
FSAM	0.0095	0.0294	0.0496	0.0005	0.0006	0.0004	0.1277	0.1325	0.1384
SEAM	0.0062	0.0280	0.0497	0.0038	0.0020	0.0003	—		
LDBM	0.0053	0.0239	0.0476	0.0047	0.0061	0.0024			
FTBM	0.0072	0.0252	0.0433	0.0028	0.0048	0.0067			

**Table 5:** The results of all test methods on subregion 1, subregion 2, and subregion 3 with spectral resolution of 9.6 nm.

**subregion 1**									
	**k**			**AE**			**MRMSE**		
	**0.01**	**0.03**	**0.05**	**0.01**	**0.03**	**0.05**	**0.01**	**0.03**	**0.05**
CC	0.0110	0.0353	0.0579	0.0010	0.0053	0.0079	0.1699	0.3075	0.2822
PC	0.0125	0.0319	0.0500	0.0025	0.0019	0.0000	0.2411	0.2399	0.2536
GM	0.0159	0.0393	0.0574	0.0059	0.0093	0.0074	0.4165	0.4669	0.4253
FSAM	0.0117	0.0306	0.0498	0.0017	0.0006	0.0002	0.1083	0.1140	0.1075
SEAM	0.0156	0.0331	0.0563	0.0056	0.0031	0.0063	—		
LDBM	0.0180	0.0343	0.0557	0.0080	0.0043	0.0057			
FTBM	0.0045	0.0219	0.0526	0.0055	0.0081	0.0026			
**subregion 2**									
	**k**			**AE**			**MRMSE**		
	**0.01**	**0.03**	**0.05**	**0.01**	**0.03**	**0.05**	**0.01**	**0.03**	**0.05**
CC	0.0066	0.0266	0.0550	0.0034	0.0034	0.0050	0.1168	0.1916	0.1896
PC	0.0102	0.0297	0.0469	0.0002	0.0003	0.0031	0.1581	0.1550	0.1596
GM	0.0096	0.0280	0.0461	0.0004	0.0020	0.0039	0.3654	0.3636	0.4190
FSAM	0.0095	0.0292	0.0501	0.0005	0.0008	0.0001	0.1190	0.1135	0.1091
SEAM	0.0058	0.0337	0.0531	0.0042	0.0037	0.0031	—		
LDBM	0.0072	0.0343	0.0573	0.0028	0.0043	0.0073			
FTBM	0.0143	0.0267	0.0438	0.0043	0.0033	0.0062			
**subregion 3**									
	**k**			**AE**			**MRMSE**		
	**0.01**	**0.03**	**0.05**	**0.01**	**0.03**	**0.05**	**0.01**	**0.03**	**0.05**
CC	0.0060	0.0259	0.0556	0.0040	0.0041	0.0056	0.1054	0.1535	0.1867
PC	0.0098	0.0299	0.0479	0.0002	0.0001	0.0021	0.1690	0.1658	0.1672
GM	0.0090	0.0307	0.0487	0.0010	0.0007	0.0013	0.3147	0.3168	0.3535
FSAM	0.0095	0.0284	0.0487	0.0005	0.0016	0.0013	0.1129	0.1113	0.1148
SEAM	0.0053	0.0333	0.0560	0.0047	0.0033	0.0060	—		
LDBM	0.0179	0.0242	0.0521	0.0079	0.0058	0.0021			
FTBM	0.0085	0.0231	0.0413	0.0015	0.0069	0.0087			

**Table 6:** The results of LSM detection on hyperspectral data acquired by FAHI.

Left FOV			Middle FOV			Right FOV		
#1	#2	#3	#1	#2	#3	#1	#2	#3
−0.0304	−0.0324	−0.0307	−0.0308	−0.0297	−0.0296	−0.0307	−0.0310	−0.0304
**Average:** −0.0306								

## Data Availability

Not applicable.
